# Superior Mesenteric Artery Syndrome: Clinical, Endoscopic, and Radiological Findings

**DOI:** 10.1155/2018/1937416

**Published:** 2018-08-27

**Authors:** Emanuele Sinagra, Dario Raimondo, Domenico Albano, Valentina Guarnotta, Melania Blasco, Sergio Testai, Marta Marasà, Vincenzo Mastrella, Valerio Alaimo, Valentina Bova, Giovanni Albano, Dario Sorrentino, Giovanni Tomasello, Francesco Cappello, Angelo Leone, Francesca Rossi, Massimo Galia, Roberto Lagalla, Federico Midiri, Gaetano Cristian Morreale, Georgios Amvrosiadis, Guido Martorana, Marcello Giuseppe Spampinato, Vittorio Virgilio, Massimo Midiri

**Affiliations:** ^1^Gastroenterology and Endoscopy Unit, Fondazione Istituto G. Giglio, Contrada Pietra Pollastra Pisciotto, 90015 Cefalù, Italy; ^2^Euro-Mediterranean Institute of Science and Technology (IEMEST), 90100 Palermo, Italy; ^3^Department of Radiology, DIBIMED, University of Palermo, Via del Vespro 127, 90127 Palermo, Italy; ^4^Section of Cardio-Respiratory and Endocrine-Metabolic Diseases, Biomedical Department of Internal and Specialist Medicine (DIBIMIS), University of Palermo, Piazza delle Cliniche 2, 90127 Palermo, Italy; ^5^Internal Medicine Unit, Fondazione Istituto G. Giglio, Contrada Pietra Pollastra Pisciotto, 90015 Cefalù, Italy; ^6^Radiology Unit, Fondazione Istituto G. Giglio, Contrada Pietra Pollastra Pisciotto, 90015 Cefalù, Italy; ^7^Department of Experimental Biomedicine and Clinical Neuroscience, Section of Human Anatomy, University of Palermo, 90100 Palermo, Italy; ^8^BioNec, Section of Histology, Department of Experimental and Clinical Neurosciences, University of Palermo, Palermo, Italy; ^9^Gastroenterology Unit, PO. V. Cervello, via Trabucco, 90146 Palermo, Italy; ^10^Surgery Unit, Fondazione Istituto G. Giglio, Contrada Pietra Pollastra Pisciotto, 90015 Cefalù, Italy; ^11^Strategic Direction, Fondazione Istituto Giuseppe Giglio, Cefalù, Italy; ^12^Division of Vascular Surgery, Garibaldi Hospital, Catania, Italy

## Abstract

**Background:**

The superior mesenteric artery (SMA) syndrome is a rare entity presenting with upper gastrointestinal tract obstruction and weight loss. Studies to determine the optimal methods of diagnosis and treatment are required.

**Aims and Methods:**

This study aims at analyzing the clinical presentation, diagnosis, and management of SMA syndrome. Ten cases of SMA syndrome out of 2074 esophagogastroduodenoscopies were suspected. A contrast-enhanced computed tomography (CECT) scan was performed to confirm the diagnosis. After, a gastroenterologist and a nutritionist personalized the therapy. Furthermore, we compared the demographical, clinical, endoscopic, and radiological parameters of these cases with a control group consisting of 10 cases out of 2380 EGDS of initially suspected (but not radiologically confirmed) SMA over a follow-up 2-year period (2015-2016).

**Results:**

The prevalence of SMA syndrome was 0.005%. Median age and body mass index were 23.5 years and 21.5 kg/m^2^, respectively. Symptoms developed between 6 and 24 months. Median aortomesenteric angle and aorta-SMA distance were 22 and 6 mm, respectively. All patients improved on conservative treatment. In our series, a marked (>5 kg) weight loss (*p* = 0.006) and a long-standing presentation (more than six months in 80% of patients) (*p* = 0.002) are significantly related to a diagnosis of confirmed SMA syndrome at CECT after an endoscopic suspicion. A “resembling postprandial distress syndrome dyspepsia” presentation may be helpful to the endoscopist in suspecting a latent SMA syndrome (*p* = 0.02). The narrowing of both the aortomesenteric angle (*p* = 0.001) and the aortomesenteric distance (*p* < 0.001) was significantly associated with the diagnosis of SMA after an endoscopic suspicion; however, the narrowing of the aortomesenteric distance seemed to be more accurate, rather than the narrowing of the aortomesenteric angle.

**Conclusion:**

SMA syndrome represents a diagnostic and therapeutic challenge. Our results show the following findings: the importance of the endoscopic suspicion of SMA syndrome; the preponderance of a long-standing and chronic onset; a female preponderance; the importance of the nutritional counseling for the treatment; no need of surgical intervention; and better diagnostic accuracy of the narrowing of the aorta-SMA distance. Larger prospective studies are needed to clarify the best diagnosis and management of the SMA syndrome.

## 1. Introduction

The superior mesenteric artery (SMA) syndrome is a rare entity, usually presenting with acute or chronic upper gastrointestinal tract obstruction and weight loss, due to the compression of the third part of the duodenum between the abdominal aorta and the SMA itself [1]. It represents an atypical cause of proximal intestinal obstruction, which occurs most frequently in young patients presenting with an important weight loss [2]. An abnormal low insertion of the SMA or a high insertion of the angle of Treitz that dislocates the duodenum to a cranial position may support this condition. Among the most frequent causes of SMA syndrome, the best recognized are an acquired anatomic abnormality occurring after scoliosis surgery, spinal trauma, abdominal surgery (e.g., total proctocolectomy and ileal J-pouch anal anastomosis), burns (since causing a hypercatabolic state), anorexia nervosa, and finally neoplastic diseases and malabsorptive states, which may be related to prolonged wasting conditions [2–6]. Patients with SMA syndrome may present both acutely and insidiously, thus making the diagnosis of the SMA syndrome as challenging and often delayed. Furthermore, the optimal treatment of SMA syndrome also remains a challenge. Indeed, after the diagnosis, conservative treatment with nutritional support and positioning should be tried first, and surgery may represent a lasting therapeutic option in case of failure. To date, few and small-number studies analyzed all these features of this syndrome, also due to the rarity of SMA syndrome. For this reason, studies to determine the optimal methods of diagnosis and treatment are essential. This study aims at analyzing the clinical presentation, diagnosis, and management of SMA syndrome.

## 2. Materials and Methods

Over a 2-year period (2013-2014), 10 cases of SMA syndrome out of 2074 esophagogastroduodenoscopies (EGDS) were initially suspected (see Table 1). In this setting, a pulsatile extrinsic compression in the third portion of the duodenum represented, to date, the most reliable finding which could guide the endoscopist to suspect SMA syndrome.

Once EGDS was performed, and once SMA syndrome was suspected after upper endoscopy, contrast-enhanced computed tomography (CECT) scan was performed on these patients to confirm the diagnosis.

CECT scan was done on a multidetector scanner with routine protocol comprising of no enhanced phase followed by arterial and portal phases performed after the administration of a bolus of 80–100 ml of nonionic iodinated contrast agent (Iohexol, Omnipaque 300, GE Healthcare, Princeton, NJ). The values for the study were obtained in the arterial phase using reformatted images: maximum intensity projection and multiplanar reconstruction with selected axial and sagittal images. In this setting, the CECT axial section should show the compression of the third part of the duodenum between the SMA and abdominal aorta, with proximal duodenal (and gastric) dilation. Furthermore, at the sagittal multiplanar reconstruction CECT, the angle between the SMA and aorta was measured at the origin. In adults, SMA usually forms an angle of 45° with the aorta, with the normal angle ranging from 25° to 60° [7], while clinical SMA syndrome manifestations appear if the angle drops below 25°. It is believed that values of this angle may be lower for pediatric patients [2].

On the other hand, the perpendicular distance between the SMA and aorta was measured at the site where the duodenum crosses between the lower border of the duodenum (D3) and the midpoint of the duodenal loop which is crossing at that site (D2). Further criteria for the diagnosis of SMA syndrome included an aortomesenteric distance of less than 8–10 mm [8], measured at the site where the duodenum crosses between D3 and D2.

Furthermore, we compared the demographical, clinical, endoscopic, and radiological parameters of these cases with a control group consisting of 10 cases out 2380 EGDS of initially suspected (but not radiologically confirmed) SMA over a follow-up 2-year period (2015-2016).

The Statistical Packages for Social Science SPSS version 17 (SPSS, Inc.) was used for data analysis. The analysis was performed at the group level. The normality of quantitative variables was tested with the Shapiro-Wilk test. Data were presented as the median and interquartile ranges for continuous variables. The Mann–Whitney test was used to compare the numerical variables in the two groups of patients (patients with SMA and controls). A *p* value < 0.05 was considered statistically significant.

Once the diagnosis of SMA syndrome was confirmed, the patients were referred to a gastroenterologist and to a nutritionist to discuss a personalized approach of therapy; furthermore, for each patient, a surgical consultation was proposed.

## 3. Results

In our series, we prospectively evaluated 10 cases of SMA (2 males, 8 females), with a prevalence of 0.005% (see Table 2). Median age was 40 years (range 14–40), and the median body mass index was 21.5 kg/m^2^. Symptoms developed between 6 and 24 months (median 18 months).

In the control group, we prospectively evaluated 10 cases of endoscopically suspected (but not confirmed through CECT) SMA (1 male, 9 females), with a prevalence of 0.003% (see Table 2).

Furthermore, in the follow-up period, we detected further 2 cases (both females) of SMA syndrome that were suspected at the EGDS and successively confirmed at CECT.

In this last group, 2 out of 10 patients refused to undergo CECT to confirm the initially suspected SMA, because they improved conservatively after, respectively, gluten avoidance (since celiac disease was concurrently suspected at the EGDS and successively confirmed at the histological analysis) and after *Helicobacter pylori* eradication. In this group, median age was 34.5 years (range 17–53), and the median body mass index was 23 kg/m^2^. Symptoms developed between 0 and 15 months (median 2.5 months).

The most common presentation in the SMA group was “postprandial distress syndrome (epigastric pain and discomfort, nausea, and vomiting) dyspepsia” (*p* = 0.02), according to Rome IV criteria, and weight loss (median weight loss before diagnosis was 6 kg), while in the control group, the most common presentation was “epigastric pain syndrome” dyspepsia (*p* = 0.01), according to Rome IV criteria, with a less marked weight loss (median weight loss before diagnosis was 0.5 kg).

**Figure 1:**
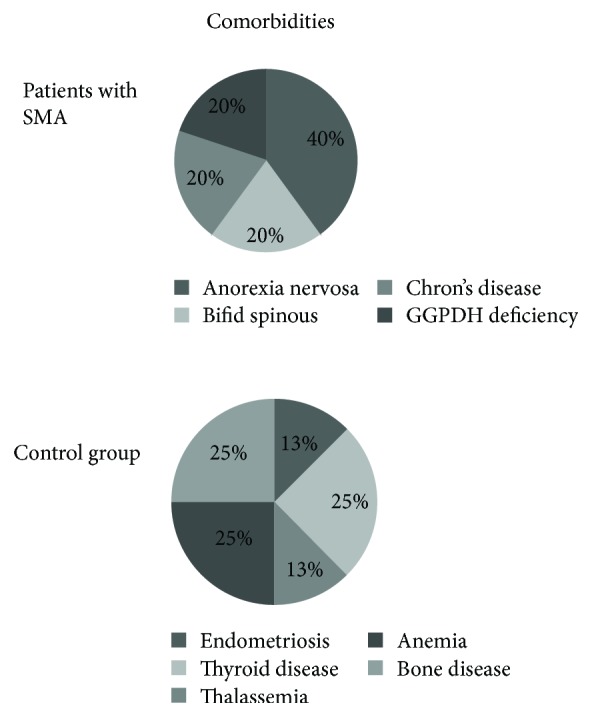
Percentage of patients with comorbidities in both groups (SMA and controls).

Premorbid conditions were present in 5 patients (anorexia nervosa in 2 patients and G6PDH deficiency, spina bifida, and Crohn's disease in 3 patients), whereas other metabolic and haematological comorbidities were observed in the control group (Figure 1). Only 2 of 10 patients of the SMA group and 1 in the control group were hospitalized, due to severe malnutrition. Median aortomesenteric angle was 22° (*p* = 0.001), and median aorta-SMA distance was 6 mm (*p* < 0.001).

With regard to the control group, both the radiological parameters were significantly associated with the diagnosis of SMA after an endoscopic suspicion.

Interestingly, all the patients improved on conservative treatment in spite of the surgical consultation proposed to each patient. Treatment strategies involved a conservative measure such as nasogastric decompression (in the two hospitalized patients) and hyperalimentation followed by oral feeding and frequent small meals, through a close clinical follow-up under the supervision of a gastroenterologist and a nutritionist.

**Figure 2:**
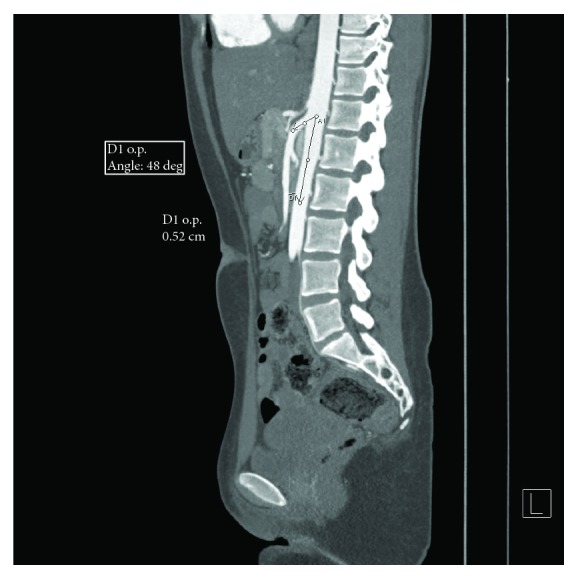
Sagittal reconstruction of a CECT scan showing the narrowing of the aortomesenteric angle and the reduction of the aorta-SMA distance (patient 1).

**Figure 3:**
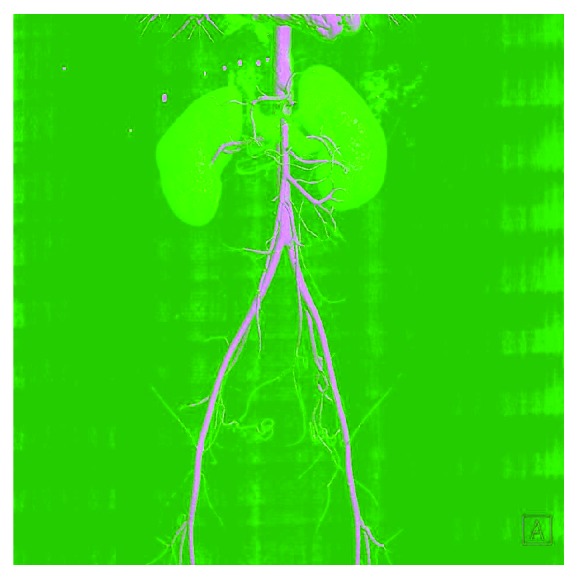
3-D angiographic reconstruction of a CECT scan showing the narrowing of the aortomesenteric angle and the reduction of the aorta-SMA distance, in the same patient (patient 1).

**Figure 4:**
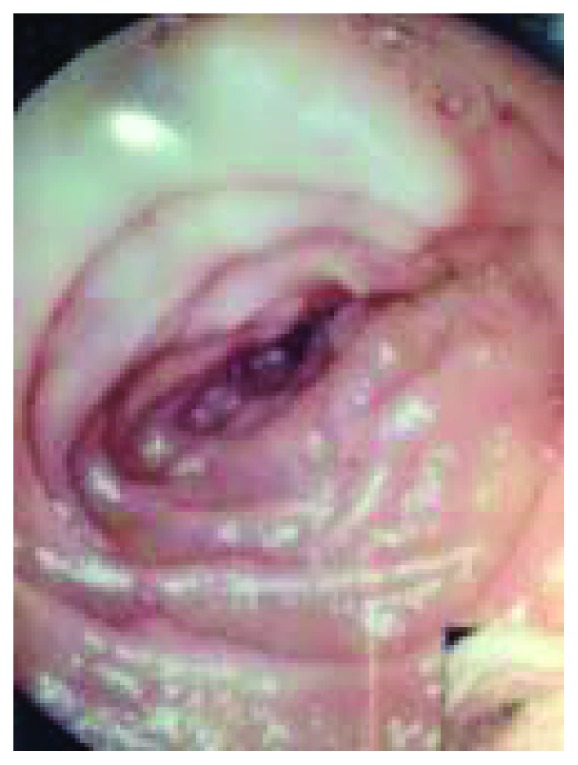
Endoscopic view showing the narrowing of the third part of the duodenum due to a pulsating extrinsic compression.

Figures 2–4 show some representative cases of our study population.

## 4. Discussion

SMA syndrome represents still a diagnostic and therapeutic challenge. Its prevalence is 0.1–0.3% [9], according to the literature arising from imaging-based studies. To our knowledge, the present study is the first that shows a prevalence of SMA syndrome based only on endoscopic findings, which could justify the relatively lower prevalence of this disease with regard to imaging studies. Interestingly, Merrett et al. published in 2009 a series of eight cases of SMA syndrome in which only one upper endoscopy suspected a possible obstruction of the third part of the duodenum [10].

Usually, SMA syndrome can present with an acute occurrence, such as a duodenal obstruction, or more insidiously, such as our patients who presented with long-standing vague abdominal pain, early satiety, anorexia, and recurrent episodes of abdominal pain associated with vomiting [11]. However, the diagnosis of the SMA syndrome is difficult and often delayed and complicated, due to its insidious presentation [1].

In our series (see Table 2), a marked (>5 kg) weight loss (*p* = 0.006) and a long-standing presentation (more than six months in the 80% of patients) (*p* = 0.002) are significantly related to a diagnosis of confirmed SMA syndrome at CECT after an endoscopic suspicion. A “resembling postprandial distress syndrome dyspepsia” presentation may be helpful to the endoscopist in suspecting a latent SMA syndrome, similar to what emerged in our study.

However, this condition affects female patients, older children, adolescents, and even underweight individuals with a history of rapid weight loss [12, 13]. In our series, we confirmed a female preponderance and a higher prevalence of the syndrome in the young-adult age group, even if there was no statistical difference with regard to age and sex ratio between the SMA group and the control group.

Upper endoscopic examination may show a pulsatile extrinsic compression indicative of this syndrome, even if only an “experienced” endoscopist may recognize this particular finding. Advances in imaging, such as in CT and magnetic resonance imaging (MRI), have dramatically helped with clear visualization of the aortomesenteric angle and of the aortomesenteric distance, thus improving the diagnostic rate [14].

CECT criteria for the diagnosis of SMA syndrome include an aortomesenteric angle of less than 22° and an aortomesenteric distance of less than 8–10 mm [8]. Usually, the aortomesenteric angle and distance significantly correlate with BMI in a normal population [15]. In our cohort, both the parameters were significantly associated with the diagnosis of SMA after an endoscopic suspicion; however, the narrowing of the aortomesenteric distance seemed to be more accurate, rather than the narrowing of the aortomesenteric angle, as diagnostic criterion for SMA syndrome, as previously suggested [16]^.^

Furthermore, both CECT and MRI are helpful to assess intra-abdominal and retroperitoneal fat [8] and to identify other problems that may require intervention, like compression of the left renal vein that results in renal vein thrombosis.

Therefore, in the appropriate clinical context, detailed history as well as endoscopic and imaging findings could raise the diagnostic yield in the case of suspicion for the diagnosis of SMA syndrome. In fact, a delay in the diagnosis can potentially lead to many complications [1].

With regard to the treatment of SMA syndrome, although many patients require surgery, in our series, all the patients were taken under close clinical follow-up by both the gastroenterologist and the nutritionist. Treatment strategies involved conservative measures such as nasogastric decompression (in the two hospitalized patients) and hyperalimentation followed by oral feeding and frequent small meals, with parallel initiation of nutritional support, prokinetics, and proton pump inhibitors. Posturing techniques at the times of meals and motility agents may be helpful in these patients [17]. The role of nutritional counseling seemed to be particularly useful in the management of our patients during the follow-up.

All the patients, despite that a surgical consultation was proposed, did not require any surgical intervention, in contrast with previous studies [18], with the exception of isolated reports [11].

All the patients underwent a close clinical follow-up under the supervision of both the nutritionist and the gastroenterologist. A further endoscopical or radiological follow-up was not proposed, since guidelines about the follow-up of SMA syndrome do not exist, due to the invasiveness of upper endoscopy (in fact, once that the diagnosis was established, we did not consider a second look by endoscopy as useful) and due to the concern inherent to the exposure to ionizing radiation (since the patients experienced a positive clinical response).

In conclusion, with regard to a previously published series, our results show the following significant aspects: the importance of the endoscopic suspicion of SMA syndrome, when confirmed by CECT scan; the preponderance of a long-standing and chronic onset; a female preponderance; the importance of nutritional counseling in the therapeutic approach; the absence of a need for surgical intervention; and the better diagnostic accuracy of the narrowing of the aorta-SMA distance, rather than the narrowing of the aortomesenteric angle. However, further prospective studies, with a larger number of patients, are needed to clarify the best way to diagnose and manage the SMA syndrome.

## Figures and Tables

**Table 1:** Demographical, clinical, endoscopic, and radiological findings of patients with superior mesenteric artery syndrome.

	Patient 1	Patient 2	Patient 3	Patient 4	Patient 5	Patient 6	Patient 7	Patient 8	Patient 9	Patient 10
Hospital admission	Not	Not	Not	Yes (14 days)	Not	Yes (12 days)	Not	Not	Not	Not
Age	34	17	14	40	23	38	23	24	25	23
Sex	Female	Female	Female	Male	Female	Female	Male	Female	Female	Female
Weight (kg)	45	84	50	45	60	40	65	43	47	43
Body mass index (kg/m^2^)	20	28	22	19	23	18	22	15	21.5	21.5
Weight loss before the diagnosis (kg)	10	5	6	10	6	20	16	5	6	5
Comorbidities	Anorexia nervosa	GP6DH deficiency	None	Crohn's disease	None	Spina bifida	None	Anorexia nervosa	None	None
Further endoscopic findings	None	*Helicobacter pylori*-related gastritis	Grade A esophagitis	Cardial incontinence	Cardial incontinence	None	Hiatal ernia; *Helicobacter pylori*-related gastritis	Cardial incontinence	Cardial incontinence	None
Onset (months)	12	12	12	6	24	18	18	6	18	18
Clinical presentation	Dismotility-like dyspepsia	Dismotility-like dyspepsia	Dismotility-like dyspepsia	Otherwise unexplained weight loss	Reflux-like dyspepsia	Otherwise unexplained weight loss	Dismotility-like dyspepsia	Dismotility-like dyspepsia	Reflux-like dyspepsia	Dismotility-like dyspepsia
Diagnosis	Upper endoscopy, abdominal computed tomography	Upper endoscopy, abdominal computed tomography	Upper endoscopy, abdominal computed tomography	Upper endoscopy, abdominal computed tomography	Upper endoscopy, abdominal computed tomography	Upper endoscopy, abdominal computed tomography	Upper endoscopy, abdominal computed tomography	Upper endoscopy, abdominal computed tomography	Upper endoscopy, abdominal computed tomography	Upper endoscopy, abdominal computed tomography
Aortomesenteric angle	23	38	15	15	46	24	20	21	22	22
Aorta-superior mesenteric artery distance	6	5	5	4	6	6	6	5	6	6
Treatment	Conservative	Conservative	Conservative	Conservative	Conservative	Conservative	Conservative	Conservative	Conservative	Conservative

**Table 2:** Characteristics of patients with SMA and control group.

Parameters	Patients with SMA (*N* = 10)	Control group (*N* = 10)	*p*
	Median (IR)	Median (IR)	
Age (years)	40 (14–65)	34.5 (17–53)	0.912
Body mass index (kg/m^2^)	22 (15–28)	23 (17–26)	0.315
Weight decrease (kg)	6 (5–20)	0.5 (0–13)	0.006
Onset of symptoms	14 (6–24)	2.5 (0–15)	0.002
Aortomesenteric angle (mm)	22 (15–46)	74.5 (25–87)	0.001
Aorta-SMA distance (mm)	6 (4–6)	11 (10–12)	<0.001
	*Subjects* (%)	*Subjects* (%)	
Gender			
Male	2 (20%)	1 (10%)	
Female	8 (80%)	9 (90%)	0.540
Hospitalization	2 (20%)	1 (10%)	0.531
Comorbidities	5 (50%)	8 (80%)	0.160
*Clinical symptoms at onset*			
Postprandial distress syndrome	7 (70%)	2 (20%)	0.025
Otherwise unexplained weight loss	2 (20%)	2 (20%)	1
Gastroesophageal reflux disease	1 (10%)	1 (10%)	1
Epigastric pain syndrome	0 (0)	5 (50%)	0.010
*Further endoscopic findings*			
*Helicobacter pylori* presence	2 (20%)	3 (30%)	0.606
Erosive gastroesophageal reflux disease	1 (10%)	2 (20%)	0.531
Cardial incontinence	4 (40%)	4 (40%)	1
Hiatal hernia	1 (10%)	4 (40%)	0.121
D-G reflux	0 (0)	2 (20%)	0.136
Celiac disease	0 (0)	1 (10%)	0.305
Gastric polyps	0 (0)	1 (10%)	0.305

## Data Availability

The data used to support the findings of this study are available from the corresponding author upon request.
